# Advancing the defensive explanation for anxiety disorders: lorazepam effects on human defense are systematically modulated by personality and threat-type

**DOI:** 10.1038/tp.2013.20

**Published:** 2013-04-16

**Authors:** A M Perkins, U Ettinger, K Weaver, A Schmechtig, A Schrantee, P D Morrison, A Sapara, V Kumari, S C R Williams, P J Corr

**Affiliations:** 1 King's College London, Department of Psychological Medicine, Institute of Psychiatry, London, UK; 2 Department of Psychology, University of Bonn, Bonn, Germany; 3 King's College London, Department of Neuroimaging, Institute of Psychiatry, London, UK; 4 Department of Radiology, Academic Medical Center, Amsterdam, The Netherlands; 5 King's College London, Department of Psychosis Studies, Institute of Psychiatry, London, UK; 6 King's College London, Department of Psychology, Institute of Psychiatry, London, UK; 7 Department of Psychology, City University London, London, UK

**Keywords:** anxiety, fear, flight, human defense, lorazepam, risk assessment

## Abstract

Clinically effective drugs against human anxiety and fear systematically alter the innate defensive behavior of rodents, suggesting that in humans these emotions reflect defensive adaptations. Compelling experimental human evidence for this theory is yet to be obtained. We report the clearest test to date by investigating the effects of 1 and 2 mg of the anti-anxiety drug lorazepam on the intensity of threat-avoidance behavior in 40 healthy adult volunteers (20 females). We found lorazepam modulated the intensity of participants' threat-avoidance behavior in a dose-dependent manner. However, the pattern of effects depended upon two factors: type of threat-avoidance behavior and theoretically relevant measures of personality. In the case of flight behavior (one-way active avoidance), lorazepam increased intensity in low scorers on the Fear Survey Schedule tissue-damage fear but reduced it in high scorers. Conversely, in the case of risk-assessment behavior (two-way active avoidance), lorazepam reduced intensity in low scorers on the Spielberger trait anxiety but increased it in high scorers. Anti-anxiety drugs do not systematically affect rodent flight behavior; therefore, we interpret this new finding as suggesting that lorazepam has a broader effect on defense in humans than in rodents, perhaps by modulating general perceptions of threat intensity. The different patterning of lorazepam effects on the two behaviors implies that human perceptions of threat intensity are nevertheless distributed across two different neural streams, which influence effects observed on one-way or two-way active avoidance demanded by the situation.

## Introduction

Anxiety disorders are the most prevalent class of psychiatric illness, affecting ∼60 million people per year in Europe alone.^1^ Moreover, personality traits that reflect individual differences in proneness to anxiety are an important risk factor for psychiatric illness in general.^2^ Studies capable of explaining the nature of anxiety, and why some people are especially prone to it, are of fundamental importance in psychiatry. One theory postulates that anxiety, as an evolved adaptation, is a defensive reaction;^3^ and that anxiety disorders reflect hyperactivity in brain systems that control defensive behavior.^4^ Specifically, it has been argued that trait individual differences in proneness to anxiety (phenotypic personality) are caused by individual differences in sensitivity to threat.^5^

To date, experimental support for the defensive explanation of anxiety disorders stem chiefly from ethopharmacological studies showing that drugs with clinical effectiveness against anxiety disorders in humans systematically alter the innate defensive behavior of rodents.^6^ Concerns that rodent models of psychological processes are too simple to apply to humans demand validation evidence in humans.^7^ Neuroimaging studies show that human brain systems, which govern defensive behavior, also generate clinically important negative emotions,^8, 9, 10^ and a candidate genetic risk factor for panic disorder has been found to potentiate flight behavior.^11^ However, data more directly comparable to the cardinal rodent findings are required, such as studies that systematically characterize the effects of drugs with clinical effectiveness against anxiety disorders on human defensive behavior.

To begin the ethopharmacological validation of the defensive explanation for fear and anxiety in humans, we previously tested the effects on intensity of threat-avoidance behavior of two drugs used to treat anxiety disorders, namely lorazepam (1 mg) and citalopram (10 mg).^12^ On the basis of the defensive direction theory, which associates anxiety and fear with activity in parallel neural streams activated by approach to threat and departure from threat, respectively,^5, 13^ we hypothesized that lorazepam (an anti-anxiety drug^14^) would alter the intensity of behavior in response to threats requiring approach (that is, two-way active avoidance). We also predicted that citalopram (an anti-panic drug^15^) would alter the intensity of behavior in response to threats that need not be approached (that is, one-way active avoidance). In 30 healthy adult male humans, we found support for the former hypothesis, as 1 mg of lorazepam modulated risk-assessment intensity (RAI), which we operationalized as the magnitude of forward–backward oscillation during approach to threat (an anxiety-related defensive behavior that is part of the rodent risk-assessment response^16^) in a human analog of the rodent paradigm, but 10 mg citalopram exerted no significant main effect on flight intensity (FI; a fear-related behavior in rodents^17^). Here, we build on these previous findings by testing the effects of two different doses of lorazepam (1 and 2 mg) on one-way and two-way active avoidance in humans.

First, congruent with the theory that anxiety is elicited by threats requiring approach and fear by threats that need not be approached,^5, 13^ we predicted that scores on a clinically inspired questionnaire measure of anxiety-proneness (trait anxiety) would correlate positively with RAI, whereas questionnaire scores on a clinically developed fear-proneness scale (trait fear) would correlate positively with FI. We additionally predicted that scores on a general (super-ordinate) measure of proneness to negative emotion (neuroticism) would correlate positively with both RAI and FI. As all three of these questionnaires gauge emotional dispositions, not behavioral outputs, there is no *a priori* reason to expect a positive correlation between any of their scores and the intensity of threat-avoidance behavior unless such emotions are, indeed, defensive in origin.

Second, anti-anxiety drug effects on rodent defensive behavior are heterogeneous, with their patterning depending upon the perceived level of threat in the situation: anti-anxiety drugs reduce RAI in rodents exposed to mild threat, but increase it in rodents exposed to severe threat.^3^ Extrapolating these rodent data to humans, it has been proposed that human (trait) personality differences are comparable to rodent experimentally induced (state) differences.^5, 13^ Thus, a human scoring high on trait anxiety is viewed as analogous to a severely threatened rodent, and vice versa. According to this argument, lorazepam should increase risk-assessment behavior in high-trait anxiety people, but decrease it in low-trait anxious people. This theory predicts an interaction of trait anxiety and lorazepam (the presence of a main drug effect would depend on the exact form of this interaction). In contrast, as anti-anxiety drugs do not systematically affect rodent flight behavior,^3^ fear questionnaire scores should not modulate lorazepam effects on human defensive responses to threats that need not be approached (that is, FI). Finally, lorazepam has sedative side effects;^18^ hence, we tested whether effects of lorazepam on human defense are explicable as sedation confounds.

## Participants and methods

Forty healthy volunteers (20 females; mean age 24.8 years, s.d.±4.1) gave written informed consent as required by the local ethics committee. Participants were medically screened by telephone, and those who passed were assessed in person by a psychiatrist to ensure they were physically healthy and had no current or past psychiatric disorders. After screening, volunteers were familiarized with the experimental tasks and then completed the Eysenck Personality Questionnaire–Revised,^19^ Spielberger Trait Anxiety Inventory^20^ and the Fear Survey Schedule (FSS).^21^ The first questionnaire was administered, as its neuroticism scale provides a general measure of proneness to negative emotion, scores on which should hypothetically relate positively to both FI and RAI. The last two questionnaires were administered in an attempt to measure variance in specific emotional responses connected to risk assessment and flight, respectively. More specifically, clear *a priori* reasons exist to indicate that trait anxiety is the best available index of individual differences in the reactivity of brain systems that control the (complex) avoidance of threats, but that require approach (for example, foraging in a field with potential predators).^5, 13^ We used the Tissue Damage subscale of the FSS as a covariate for FI, as there are strong *a priori* reasons for believing this construct is the best available index of individual differences in the reactivity of brain systems that control (simple) avoidance of threats that need not be approached.^11^

We used a double-blind, placebo-controlled, randomized, within-subjects design, comprising three experimental sessions: placebo (50 mg ascorbic acid), and 1 mg and 2 mg lorazepam in a randomized order, and scheduled a week apart to allow drug washout. Drugs were administered with 300 ml of water and were contained in opaque capsules so that the participants and experimenters were blind to the experimental condition. After a 2-h wait for drug metabolism, volunteers completed the experimental session that lasted ∼40 min.

**Figure 1:**
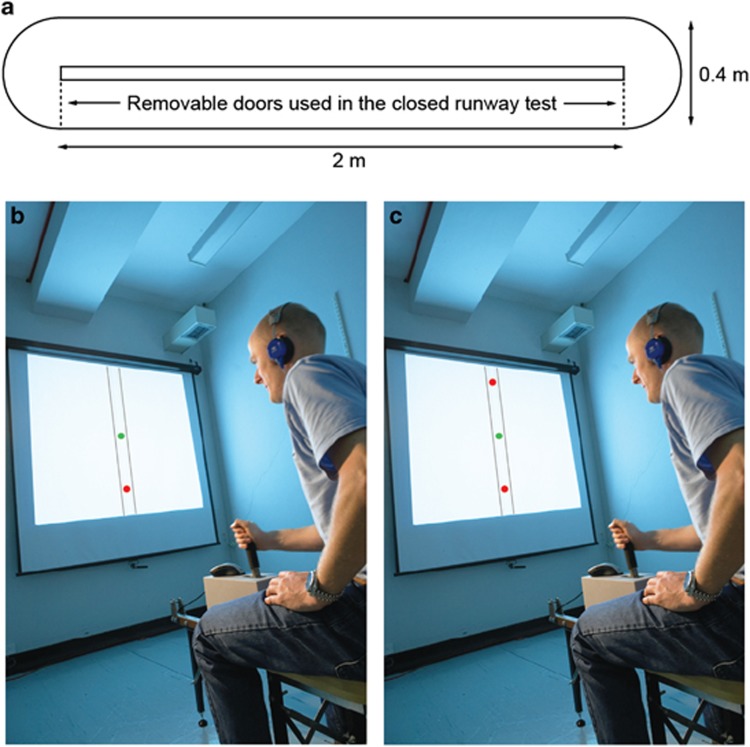
(**a**) The Mouse Defense Test Battery (MDTB). (**b**, **c**) The human translation of the MDTB, the Joystick Operated Runway Task. A force-sensing joystick apparatus (PH-JS1; Psyal, London, UK) controls the speed of a cursor (green dot) in an on-screen runway; the harder the joystick is pushed, the faster the cursor travels. In the one-way active avoidance phase, this cursor was pursued by a single threat stimulus (red dot; **b**). Participants received an unpleasant but harmless 115-dB white noise burst of near instantaneous rise time lasting 250 ms if the red dot collided with the green dot. The two-way active avoidance phase (**c**) was identical, except that a second red dot travelled ahead of the green dot at a constant velocity, causing a goal conflict whereby the participant had to travel fast enough to avoid the pursuing threat, but not so fast that they collided with the leading threat stimulus.

The intensity of threat-avoidance behavior was indexed using the Joystick Operated Runway Task (JORT^11^), a human translation of the Mouse Defense Test Battery (Figure 1a^22^), which measures the one-way active avoidance (labeled FI Figure 1b) and two-way active avoidance (labeled RAI; Figure 1c) in response to threat of a 115-dB white noise burst. To control for individual differences in the participants' motor function and sedation effects of lorazepam, responses were measured with and without threat, as signaled by the presence or absence, respectively, of a lightning flash icon on screen. In the Mouse Defense Test Battery, escape speed is a measure of rodent FI;^23^ in the human translation of the task,^12^ FI related to the degree to which threat (as signaled by the lightning flash icon) increased the velocity of the green dot cursor along the runway during one-way avoidance of the red dot cursor, as shown in Figure 1b (that is, average velocity in the one-way active avoidance trials that contained no threat of white noise subtracted from the average velocity in the one-way active avoidance trials with a threat of white noise).

In the Mouse Defense Test Battery, approach-withdrawal oscillation in the closed runway configuration is a component of rodent risk-assessment behavior.^16^ In the same task, approach-withdrawal oscillation has been linked to anxiety by the finding that this behavior is sensitive to anxiolytic drugs.^17^ When the task was translated for human use,^12^ RAI was the label chosen to describe the degree to which threat (as signaled by the lightning flash icon) increased the magnitude of forward–backward oscillation of the green dot when trapped between the two red dot cursors (as shown in Figure 1c).^12^ The face validity of the label of ‘RAI' is limited, as in the human version of the task the forward–backward oscillation serves no information-gathering function. Nevertheless, to remain consistent with the previously published research,^12^ the label was retained in the present experiment with the proviso that the forward–backward oscillation, labeled as risk assessment, should be more strictly likened to the hesitant oscillation behavior that has been noted for decades as a behavioral marker of goal conflict in rodents, regardless of whether or not it gathers information.^5^ RAI in the JORT was accordingly calculated as standard deviation (s.d.) of the average velocity (V) in the two-way active avoidance trials that contained no threat (Ta) of white noise subtracted from s.d. of the average velocity in the two-way active avoidance trials with threat (Tp) of white noise. Thus, RAI=V(s.d.).Ta−V(s.d.).Tp.

Each testing session consisted of 48 trials (12 of each of the above types) presented in a pseudo-random order to enhance unpredictability. To enhance further unpredictability, intertrial intervals were varied pseudo-randomly between 15 and 30 s. To prevent prolonged exposure to white noise, a trial terminated automatically as soon as the participant had received a burst of white noise. If the participant successfully avoided the threat stimuli for 7 s, the trial automatically terminated.

To assess the effect of individual differences in how aversive the participants found the JORT, a questionnaire measure of state affect (the short-scale Positive Affect and Negative Affect Schedule^24^) was administered immediately before and after the first completion of the task during screening. Task aversiveness was calculated as PANAS Negative score (post-task)−PANAS Negative score (pre-task).

To dissociate putative effects of lorazepam on threat-avoidance behavior from its sedative side effects,^17^ we also measured its effects on prosaccade peak velocity using an Eyelink 1000 eye-tracker (SR Research, Mississauga, ON, Canada). This task indexes the speed to track a dot. It is a sensitive and objective behavioral marker of the sedative effects of benzodiazepines (the slower the eye movement, the greater the sedation^25, 26^), but does not expose participants to threat.

Associations between dependent variables and scores on trait anxiety and fear were assessed using Pearson's product–moment correlation coefficient (SPSS v.18.0, IBM Corporation, Somers, NY, USA). Effects of drug upon the intensity of threat-avoidance behavior were analyzed by repeated measures analysis of covariances, in which drug (placebo, and 1 and 2 mg lorazepam) formed a three-level within-subjects factor. As females tend to be significantly more vulnerable than males to anxiety disorders,^27, 1^ participant sex was entered into the analysis of covariances as a between-subjects factor. The personality variables predicted to be important by the defensive direction theory (trait anxiety and tissue-damage fear) were entered as covariates in the analysis of covariances of RAI and flight intensity, respectively. Simple contrasts were used to test for the specific direction of drug effects against placebo.

## Results

### Personality effects on task performance

Table 1 shows means, s.d. and intercorrelations of individual differences and performance variables. In terms of correlations with defensive behavior, bivariate correlations showed that whereas trait anxiety and tissue damage were both significantly related to FI in placebo, only trait anxiety was significantly correlated with risk assessment intensity in the 2 mg condition. FSS tissue-damage fear was the only personality questionnaire variable that showed a statistically significant positive correlation with ratings of task aversiveness.

### Drug effects on task performance

When drug effects on task performance were analyzed without taking into account personality effects, there were no significant main effects of lorazepam on FI or RAI: *F* (2, 38)=0.1, *P*=0.922, *n*_p_^2^=0.002; *F* (2, 38)=0.1, *P*=0.903, *n*_p_^2^=0.003. Nor were there any significant drug × sex interactions for FI and RAI: *F* (2, 38)=0.3, *P*=0.744, *n*_p_^2^=0.002; *F* (2, 38)=0.5, *P*=0.638, *n*_p_^2^=0.012. However, when personality variables were included in the analysis as covariates, significant modulating effects of the drug on defensive behavior were found.

**Figure 2:**
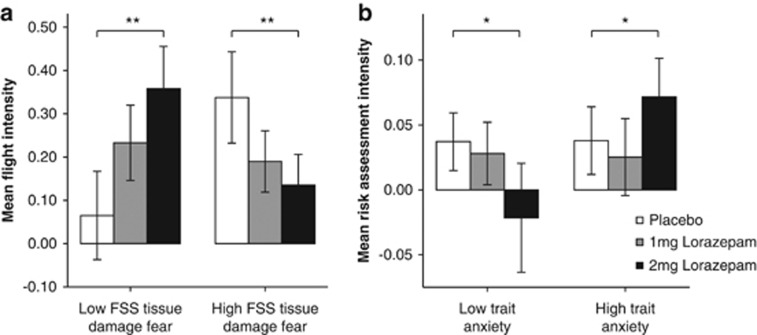
(**a**) Flight intensity (FI) was significantly decreased by lorazepam in participants with high levels of threat magnification, as indexed by the tissue-damage subscale of the Fear Survey Schedule (FSS), but FI was increased in low FSS scorers. (**b**) Risk assessment intensity (RAI) was significantly higher in participants scoring above the median on Trait Anxiety (error bars represent one s.e.m.; *P<0.05, **P<0.01). It should be noted that the division of the groups into high–low scorers was purely for illustrative purposes; the analysis of covariance was conducted using questionnaires as continuous variables.

With regard to RAI, the main effect of lorazepam reached trend level significance, *F* (2, 36)=2.5, *P*=0.086, *n*_p_^2^=0.064, as did the drug by trait anxiety interaction, *F* (2, 36)=2.5, *P*=0.089, *n*_p_^2^=0.063. However, simple contrasts revealed that for the placebo versus 2 mg lorazepam condition, there was a significant effect of drug, *F* (1, 39)=4.3, *P*=0.045, *n*_p_^2^=0.105, and also a significant interaction between lorazepam and trait anxiety questionnaire scores: *F* (1, 39)=4.2, *P*=0.049, *n*_p_^2^=0.101. In the placebo versus 1 mg condition, there were no significant effects of drug, *F* (1, 39)=0.2, *P*=0.642, *n*_p_^2^=0.006 or of drug with trait anxiety, *F* (1, 39)=0.1, *P*=0.710, *n*_p_^2^=0.004. Figure 2b illustrates this interaction by dividing the sample along the median on trait anxiety, showing that RAI was reduced by 2 mg lorazepam in low scorers on trait anxiety but was increased in high scorers on trait anxiety. There was no significant interaction between drug condition and participant sex, *F* (2, 74)=0.5, *P*=0.595, *n*_p_^2^=0.014, nor was there a significant interaction between subjects effects on RAI of participant sex, *F* (1, 37)=0.0, *P*=0.971, *n*_p_^2^=0.000, or trait anxiety, *F* (1, 37)=1.5, *P*=0.222, *n*_p_^2^=0.040.

Contrary to expectations, lorazepam altered FI, showing a significant main effect of drug condition, *F* (2, 36)=5.4, *P*=0.006, *n*_p_^2^=0.127, no significant interaction between drug condition and participant sex, *F* (2, 36)=0.6, *P*=0.539, *n*_p_^2^=0.017, but a significant interaction between drug condition and scores on FSS tissue-damage fear, *F* (2, 36)=6.4, *P*=0.003, *n*_p_^2^=0.148. Figure 2a reveals that lorazepam decreased FI in participants in the upper half of the sample on tissue-damage fear (that is, the particularly threat-sensitive participants) but increased it in the lower half of the sample (that is, the particularly threat-insensitive participants). This outcome was confirmed by simple contrasts between placebo and 2 mg lorazepam: *F* (1, 39)=8.6, *P*=0.006, *n*_p_^2^=0.189. The contrast for placebo versus 1 mg failed to reach significance: *F* (1, 39)=2.6, *P*=0.110, *n*_p_^2^=0.068. A similar pattern emerged for the simple contrasts for the interaction of drug with FSS tissue-damage fear (placebo versus 1 mg: *F* (1, 39)=3.5, *P*=0.068, *n*_p_^2^=0.068; placebo versus 2 mg *F* (1, 39)=10.2, *P*=0.003, *n*_p_^2^=0.216. There was also a main effect of sex, indicating that FI was significantly higher in females than males, irrespective of drug condition or personality questionnaire scores, *F* (1, 37)=5.0, *P*=0.031, *n*_p_^2^=0.119. The between subjects effect of FSS tissue-damage fear on FI failed to reach significance, *F* (1, 37)=2.3, *P*=0.134, *n*_p_^2^=0.060.

### Drug effects on prosaccade peak velocity

Repeated measures analysis of variance showed a large and significant main effect of drug condition on prosaccade peak velocity, *F* (2, 37)=28.1, *P*<0.001, *n*_p_^2^=0.425, with simple contrasts revealing that, as expected, lorazepam reduced prosaccade peak velocity in a dose-dependent manner: placebo versus 1 mg: *F* (1, 38)=21.4, *P*<0.001, *n*_p_^2^=0.360; placebo versus 2 mg; *F* (1, 38)=43.2, *P*<0.001, *n*_p_^2^=0.532. Table 2 shows correlations between the changes (that is, drug score–placebo score) induced by lorazepam in prosaccade peak velocity and the changes induced by lorazepam in the JORT variables of FI and risk assessment. There were no significant correlations between these two forms of lorazepam effects, indicating that the sedative effect of lorazepam was not responsible for altering the defensive behavior of participants.

## Discussion

We found two forms of support for the general hypothesis that anxiety-related illness is caused by alterations in the functioning of brain systems that control defensive behaviour.^3^ First, scores on clinically relevant questionnaire measures of fear and anxiety proneness were positively correlated with the intensity of flight and risk-assessment behavior, respectively (Table 1). Second, these questionnaire scores differentially modulated the effects of lorazepam on these two defensive behaviors.

The patterning of the interactive drug and personality effects on defensive behavior was complex, but those pertaining to RAI conformed to the predictions of the defensive direction theory.^5, 13^ This theory associates anxiety with threats that require approach and fear with threats that need not be approached. This theory postulates that these two threat types are processed by two parallel defensive systems. The theory maintains that trait individual differences in the reactivity of the two systems influence an individual's personality profile. More specifically, it has been predicted that individuals with particularly high levels of reactivity in the systems controlling responses to threats that require approach theoretically should display high scores on trait anxiety.^13^

This is important in the present context, as the theory likens individual differences in human-trait anxiety to state differences in threat exposure in rodents, and these rodent-state differences modulate anti-anxiety drug effects (anti-anxiety drugs reduce RAI in rodents exposed to mild threat, but increase it in rodents exposed to severe threat).^3^ If this aspect of the defensive direction theory is correct, in humans lorazepam should hypothetically dampen RAI in low scorers on trait anxiety, but potentiate RAI in high scorers on trait anxiety. As Figure 2b shows, this is what we found, although only for 2 mg lorazepam, suggesting that 1 mg was not a sufficiently large dose to cause systematic changes in risk assessment.

As shown in Figure 2a, effects of personality/lorazepam modulation were also found on FI. These effects are not readily explicable by the defensive direction theory, which aligns fear with flight, and hence predicts that lorazepam, as an anti-anxiety drug, should not affect FI. Nevertheless, the effects are clear: FI was reduced in a dose-dependent manner by lorazepam in high scorers on tissue-damage fear, but it was increased in a dose-dependent manner in low scorers on this scale.

Although this result differs from rodent findings, it is readily explicable in terms of the difference in the human analog of the rodent defensive situation; in relation to human defensive behavior, the context is important.^28^ In this context, there is a difference in knowledge between rodent and human subjects in experimental defensive situations. Whereas rodents are threat-naive before the task onset,^3^ our participants knew that the task entailed punishment. Thus, the entire testing session is, in effect, an approach-to-threat situation that, according to the defensive direction theory, should elicit anxiety and thus should show lorazepam effects on flight behavior. This interpretation has the merit of fitting with a theory that sustained apprehension, such as occurs in a 20-min long punishment-related testing session of the type experienced by our participants, is related to anxiety rather than fear.^29^

As regards the different patterning of effects of lorazepam and personality questionnaire scores on FI and RAI (Figures 2a and b), a viable, if tentative, explanation draws upon a supplementary postulate of the defensive direction theory, namely that within both fear- and anxiety-related neural streams, mild threats activate the upper levels, whereas severe/immediate threats activate the lower levels.^13^ As rodent studies indicate that anti-anxiety drugs reduce the perceived intensity of threat,^30^ this facet of the defensive direction theory implies that lorazepam should affect activity in both neural streams. The notion that lorazepam modulates perceptions of intensity of threats in general is backed up in humans by a variety of other studies, such as the finding that anti-anxiety benzodiazepine drugs reduce fear/anxiety-potentiated startle, but have no effect on baseline startle^31, 32^ or on pleasure-attenuated startle.^33^ However, based on our new data, we suggest the particular neural stream activated depends upon the trial type of the joystick task: the differences seen in lorazepam effects on FI and RAI (Figures 2a and b) provide circumstantial evidence for the effects of two such neural streams. This a highly theoretical conjecture, but at this early stage of understanding the neural control of human defence we hope even tentative theoretical links, such as those identified here, may provide guidance for subsequent more detailed research efforts.

Ultimately, it may be the case that our results are best explained by a combination of the two arguments we have outlined. At the mild levels of general threat experienced when the participant enters the testing chamber, it seems likely that forebrain-mediated anxiety roughly equivalent to sustained apprehension will predominate. Then, once the task is underway and threat levels peak, lower levels of the neural streams come online that are more sharply differentiated according to whether the threat stimulus must be approached. Thus, we tentatively suggest that, in humans the upper (mild threat) levels of the two neural streams are merged in favor of anxiety by the sheer generality of mild threat situations. However, once threats become more immediate, then the lower, more situationally specific levels of the neural streams become active, giving rise to the different patterning of lorazepam effects seen in flight and risk assessment. This explanation is consistent with the significant comorbidity seen among anxiety and fear disorders.^34^

The above explanation is reinforced by our finding of theoretically congruent personality effects on the two types of defensive behavior. In the case of flight behavior, lorazepam increased intensity in low scorers on FSS tissue-damage fear but reduced it in high scorers. In the case of risk assessment, lorazepam reduced intensity in low scorers on the Spielberger trait anxiety but increased it in high scorers. The validity of this latter result is supported by its conformity with our previous finding^12^ of a tendency for lorazepam to reduce RAI in low scorers on FSS social fear.

Individual differences in the general perception of threat intensity have been aligned with scores on the major personality dimension of neuroticism.^5^ This dimension is found in all of the leading descriptive models of personality and reflects individual differences in proneness to negative emotions of all kinds,^35, 36^ whether abstract or situationally elicited. In the Eysenck Personality Questionnaire–Revised neuroticism scale, the questions do not reference specific situations, but instead are phrased in abstract terms such as ‘Does your mood often go up and down?,' ‘Do you ever feel ‘just miserable' for no reason?' or ‘Would you call yourself a nervous person?.' In the light of our results, it is plausible that these questions index the effects of the merged upper levels of the fear and anxiety systems. Specifically, as most threats actually encountered in everyday life are mild, comorbidity of anxiety disorders^37^ might be explained by this combined effect, which itself is related to the fact that negative emotions in humans are usually of an anxiety nature. In contrast, it might be the case that the dedicated questionnaire measures of fear- and anxiety proneness that modulated lorazepam effects (Figures 2a and b) are indexing individual differences in reactivity, specifically at lower levels of the neural streams, but that these lower levels of the defensive system are only activated relatively infrequently in everyday life.

Finally, interpretation of our results may be informed by some highly tentative attempts to align them with certain key findings pertaining to panic and anxiety. First, although lorazepam is primarily viewed as an anti-anxiety drug, it has been found to have clinically significant anti-panic effects at doses approximately double of those at which it shows anti-anxiety effects (for example, 7 mg per day).^38, 39, 40^ Therefore, it is possible that the capacity of lorazepam to alter FI in the JORT reflects high sensitivity of the JORT to this anti-panic effect; future research could explore this possibility by testing participants on the JORT using higher doses of lorazepam, providing they did not become so sedated as to be unable to manipulate the joystick effectively. Furthermore, there is evidence that relieving anxiety can induce panic.^41^ This phenomenon is typically explained by the association of anxiety with the activation of forebrain structures that process responses to mild, complex or abstract threats. These structures are thought to inhibit activity in midbrain structures that control flight behavior and mediate panic in response to close, intense, concrete threats.^42^ Thus, one effect of an anti-anxiety drug, such as lorazepam, is to reduce inhibitory power of the forebrain, freeing the midbrain to unleash panic-based flight behavior. The boosting effect of lorazepam on FI in low scorers on tissue damage fear might tentatively be explained as reflecting this releasing effect, on the basis that some of the variance captured by this questionnaire may reflect individual differences in forebrain inhibition. This may mean that low scorers on tissue-damage fear, having relatively little forebrain inhibition, are more easily pushed into intense, panic-related flight from threat by the releasing effects of lorazepam than high scorers, even though tissue-damage fear scores are positively correlated with flight in the placebo condition (Table 1, Figure 2a).

Turning to the effects of lorazepam on RAI (Figure 2b) and links to the general construct of neuroticism, rodent studies show that benzodiazepine drugs have a greater conflict-reducing effect on nonemotional rats compared with emotional rats.^43, 44^ The two strains of rats were bred for differences in the number of fecal boluses deposited during 2 min in the open-field test.^45^ Rodents innately seek dark, sheltered, quiet areas, as these offer protection from predators, and hence the brightly lit, noisy open-field arena is highly aversive to the average rat; the number of fecal boluses produced was viewed as a marker of fear. Subsequently, it has been found that the emotional rats show greater responses on a whole range of other negative and conflict-related tasks^46^ and this has led to rats from the emotional strain being viewed as roughly analogous to a human with a highly anxiety-prone, neurotic personality.^5^ In the light of this analogy, our finding that 2 mg lorazepam significantly reduced conflict-related behavior (RAI) in low scorers on trait anxiety, yet boosted it in high scorers, would seem to be broadly consistent with findings on the effects of benzodiazepine drugs on emotional versus non-emotional rats, if we accept that trait anxiety and neuroticism are closely related in psychometric terms (correlated 0.825, Table 1).

In conclusion, we associate defensive behavior with the clinically important negative emotions of anxiety and fear, consistent with the hypothesis that anxiety disorders can be explained as reflecting changes in the functioning of defensive brain systems.^3^ Our findings also show a partial fit with the defensive direction theory^5, 13^ that aligns fear with departure from threat and anxiety with approach to threat; trait anxiety questionnaire scores were positively associated with RAI during approach to threat, and fear questionnaire scores were associated with flight behavior. Contrary to expectations, we found the anti-anxiety drug lorazepam affected both forms of behavior, but showed a differing pattern of effects on risk assessment and flight, suggesting that the defensive behavior of our participants reflected a combination of fear and anxiety activation. We speculate that initial impressions of threat elicited by the testing scenario may be processed at a merged, higher neural level, but that during the task separable neural streams governing anxiety-mediated risk assessment or fear-mediated simple avoidance also come into play, depending upon whether the threat stimulus requires approach. Our data additionally shed light on the basis of human personality more generally, as we also found a significant positive association between FI and questionnaire scores on neuroticism. This latter result agrees with the notion that individual differences in sensitivity to threat contribute variance to neuroticism^5^ and has broad relevance, as it allows our human defense findings to be fitted directly into the standard rubric of personality research.

## Figures and Tables

**Table 1:** Means, s.d. and intercorrelations of individual differences variables

*Variable*	*Mean (s.d.)*	*1*	*2*	*3*	*4*	*5*	*6*	*7*	*8*	*9*	*10*	*11*
1. Trait anxiety	35.78 (9.19)	—										
2. Tissue-damage fear	19.30 (11.45)	0.344*	—									
3. Social fear	29.65 (20.09)	0.607**	0.666**	—								
4. Neuroticism	9.15 (7.18)	0.825**	0.258	0.529**	—							
5. Task aversiveness	0.45 (2.26)	0.083	0.376*	0.117	0.084	—						
6. Flight intensity (placebo)	0.21 (0.48)	0.374*	0.421**	0.220	0.314*	0.212	—					
7. Flight intensity (1 mg lorazepam)	0.21 (0.35)	0.147	0.133	0.335*	0.313*	0.212	0.192	—		-		
8. Flight intensity (2 mg lorazepam)	0.24 (0.39)	−0.163	−0.263	−0.288	−0.139	−0.119	−0.191	0.074	—			
9. Risk assessment intensity (placebo)	0.04 (0.11)	−0.080	0.172	0.201	−0.010	0.133	−0.179	0.134	−0.177	—		
10. Risk assessment intensity (1 mg lorazepam)	0.03 (0.12)	0.013	0.152	0.121	0.188	0.047	0.003	0.073	0.023	0.034	—	
11. Risk assessment intensity (2 mg lorazepam)	0.03 (0.17)	0.318*	0.217	0.209	0.239	−0.025	0.093	0.067	−0.152	0.025	−0.051	—

*N*=40 (20 male). Correlations reflect Pearson's product–moment correlation coefficients. *P<0.05; **P<0.01.

**Table 2:** Means, s.d. and intercorrelations of changes to defensive behavior and eye movements induced by lorazepam

*Variable*	*Mean (s.d.)*	*1*	*2*	*3*	*4*	*5*	*6*
1. Change in prosaccade peak velocity (1 mg lorazepam–placebo)	−25.35 (34.13)	—					
2. Change in prosaccade peak velocity (2 mg lorazepam–placebo)	−41.65 (39.59)	0.677**	—				
3. Change in flight intensity (1 mg lorazepam–placebo)	0.02 (0.54)	0.053	0.253	—			
4. Change in flight intensity (2 mg lorazepam–placebo)	0.03 (0.67)	−0.023	0.183	0.677**	—		
5. Change in risk assessment intensity (1 mg lorazepam–placebo)	−0.01 (0.16)	−0.149	−0.216	−0.134	−0.009	—	
6. Change in risk assessment intensity (2 mg lorazepam–placebo)	−.0.01 (0.20)	−0.029	−0.057	−0.169	−0.146	0.309	—

*N*=40 (20 male). Correlations reflect Pearson's product–moment correlation coefficients *P<0.05; **P<0.01.
